# Telenursing in mental health: effect on anxiety symptoms and alcohol consumption during the COVID-19 pandemic*

**DOI:** 10.1590/1518-8345.6172.3933

**Published:** 2023-06-02

**Authors:** Divane de Vargas, Erika Gisseth León Ramírez, Caroline Figueira Pereira, Sheila Ramos de Oliveira

**Affiliations:** 1 Universidade de São Paulo, Escola de Enfermagem, São Paulo, SP, Brasil.; 2 Universidade de Guarulhos, Guarulhos, SP, Brasil.; 3 Becaria de la Coordenação de Aperfeiçoamento de Pessoal de Nível Superior (CAPES), Brasil.

**Keywords:** Telenursing, Anxiety, Alcohol Drinking, Primary Prevention, Primary Health Care, COVID-19, Teleenfermería, Ansiedad, Consumo de Bebidas Alcohólicas, Prevención Primaria, Atención Primaria de Salud, COVID-19, Telenfermagem, Ansiedade, Consumo de Bebidas Alcoólicas, Prevenção Primária, Atenção Primária à Saúde, COVID-19

## Abstract

**Objective:**

to investigate the effect of a remote intervention on anxiety symptoms and alcohol use in users of the Primary Health Care service.

**Method:**

a quasi-experimental study conducted with 1,270 participants who answered the Alcohol Use Disorders Identification Test and the State-Trait Anxiety Inventory-6. Of these, 1,033 interviewees scored for moderate/severe anxiety symptoms (STAI-6 > 3) and moderate/severe risk alcohol use (AUDIT-C > 3), and received the interventions via telephone calls with follow-up periods lasting seven and 180 days. For data analysis, a mixed-effects regression model was used.

**Results:**

the effect of the intervention performed was positive in reducing anxiety symptoms between T_0_ and T_1_ (µ=1.6, *p*<0.001) and in reducing the alcohol use pattern between T1 and T3 (µ=1.57, *p*<0.001)

**Conclusion:**

the follow-up results suggest a positive effect of the intervention in reducing anxiety and the alcohol use pattern, which tends to be maintained over time. There is diverse evidence that the intervention proposed can be an alternative for preventive care in mental health, in situations where accessibility of the user or the professional is compromised.

## Abstract (es)

**Objetivo:**

investigar el efecto de una intervención a distancia sobre los síntomas de ansiedad y el consumo de alcohol en usuarios del servicio de Atención Primaria de Salud.

**Método:**

estudio cuasiexperimental, realizado con 1270 participantes que respondieron al *Alcohol Use Disorders Identification Test* y al *State-Trait Anxiety Inventory 6*. De estos, 1033 encuestados obtuvieron puntajes de síntomas de ansiedad moderada/grave (STAI-6 > 3) y consumo de alcohol de riesgo moderado/grave (AUDIT-C>3), y recibieron las intervenciones mediante llamada telefónica con un seguimiento de siete y 180 días. Para analizar los datos se utilizó un modelo de regresión de efectos mixtos.

**Resultados:**

el efecto de la intervención realizada fue positivo para reducir los síntomas de ansiedad entre T0 y T1 (µ=1,6 p<0,001) y para reducir el patrón de consumo de alcohol entre t1 y t3 (µ=1,57 p< 0,001).

**Conclusión:**

los resultados del seguimiento sugieren que la intervención tuvo un efecto positivo en la reducción de la ansiedad y el patrón de consumo de alcohol que tiende a mantenerse en el tiempo. Existe evidencia de que la intervención propuesta puede ser una alternativa para la atención preventiva en salud mental, en situaciones donde se comprometa la accesibilidad del usuario o del profesional.

## Abstract (pt)

**Objetivo:**

investigar o efeito de uma intervenção remota nos sintomas de ansiedade e no uso de álcool em usuários do serviço de Atenção Primária à Saúde.

**Método:**

estudo quase experimental, realizado com 1270 participantes que responderam o *Alcohol Use Disorders Identification Test* e a *State-Trait Anxiety Inventory 6.* Desses, 1033 entrevistados pontuaram para sintomas de ansiedade moderada/severa (STAI-6>3) e uso de risco de álcool moderado/severo (AUDIT-C>3), e receberam as intervenções via chamada telefônica com *follow-up* de sete e 180 dias. Para análise dos dados utilizou-se um modelo de regressão de efeitos mistos.

**Resultados:**

o efeito da intervenção realizada foi positivo na redução dos sintomas de ansiedade entre T0 e T1 (µ=1,6 *p*<0.001) e na redução do padrão de uso de álcool entre t1 e t3 (µ=1,57 *p*<0.001).

**Conclusão:**

os resultados do *follow-up* sugerem efeito positivo da intervenção na redução da ansiedade e no padrão de uso de álcool, o qual tende a se manter ao longo do tempo. Há evidências de que a intervenção proposta pode ser uma alternativa para o cuidado preventivo em saúde mental, em situações nas quais a acessibilidade do usuário ou do profissional encontram-se comprometidas.

**Highlights:**

**(1)** A remote intervention with a positive impact on reducing anxiety and alcohol use.

**(2)** Nursing as a protagonist of preventive care in mental health

**(3)** A low-cost intervention that covers several population groups.

**(4)** Telenursing in mental health as a care strategy during COVID-19.

## Introduction

The COVID-19 (Coronavirus disease 2019) pandemic was declared in Brazil on March 11^th^, 2020, forcing millions of people into extended isolation and social distancing periods and to live with fear, loss of loved ones and social issues such as unemployment and loss of income^(1)^. Concomitantly with progression of the pandemic, mental health problems such as anxiety, depression and substance abuse symptoms also advanced, aggravating a problem that had already been progressively occurring in Brazil, mainly among the most vulnerable population that already lived with high violence, racism and poverty rates, which contribute to mental illness^(2)^.

The results of studies carried out in the country during the COVID-19 pandemic showed that more than 50% of the population had high levels of anxiety and depressive symptoms, as well as increased alcohol consumption during the first year^(3-4)^, suggesting an increase in the deterioration of Brazilians’ mental health during this period. According to experts, the potential long-term effects on mental health that will emerge after the health crisis can result in the emergence of severe psychiatric pathologies and in an increased need to receive specialized medical care^(5)^.

This phenomenon can represent a major challenge for governments, health professionals and researchers from low-income countries, including Brazil, given its political and socioeconomic conditions and the deficit of mental health care services available to the population even before the pandemic period^(5-7)^. Thus, the search for preventive strategies and mental health support quickly and remotely during these periods when face-to-face care is not possible should be considered in order to mitigate the possible repercussions of the pandemic on the mental health of the population.

Thus, in order to provide continuity to health services during the pandemic scenario, the use of digital platforms such as Telehealth was adopted. “Tele” is a prefix meaning “at a distance”, and is used in terms such as “telescope” or “telemetry”. When combined with the term “scope”, the prefix “tele” means an instrument to see phenomena from a distance^(8)^. Therefore, Telehealth is intrinsically associated with the incorporation of information and communication technologies into health systems^(9)^, with the use of telecommunication technologies to support remote health care, as well as education for patients and professionals^(8)^. Telenursing is a subset of Telehealth in which the focus is on the specific Nursing practice^(9)^.

Although technology use changes the means of providing Nursing care and may require skills related to its use, the Nursing Process and the practice scope do not differ in Telenursing. Nurses continue to evaluate, plan, intervene and reassess the Nursing care results, using low-tech (telephones) and high-tech technologies (computers, videoconferences, Internet, telemonitoring devices)^(10-11)^, and, more recently, resorting to communication apps such as Instagram, WhatsApp and Telegram to enable care continuity and provide services continuously^(12)^.

Although Telenursing has been used for some time in different care areas in various parts of the world, the emergence of the COVID-19 pandemic boosted use of this technology and its leverage by nurses^(13)^, who needed to employ this strategy in order to maintain continuity of the care provided, mainly to people with chronic conditions^(10,12)^, when social distancing resulted in the closure of several health services and in the reallocation of services and teams to face the demands of the pandemic, proving to be capable of mitigating these difficulties by redesigning health practices and improving the quality of care provision during this period^(14)^.

Telenursing can be an important tool to provide Nursing care in mental health, both mitigating the effects of the pandemic in preventing worsening of conditions and symptoms triggered by it and in monitoring the mental health of the population after the pandemic period.

Although Telenursing can make use of the various so-called high-tech tools currently available, such as video calls that require using a computer with a camera and Internet access or smartphones, telephone calls using low-tech (telephone device) constitute a valuable resource, mainly in places with a high digital poverty level, as is the case in Brazil, where one out of four people does not have Internet access. This represents nearly 30% of the Brazilians living in large cities and 60% in rural areas that do not access the network. On the other hand, mobile phone access is found in 93.2% of the households in urban areas and 70% in rural areas of the country^(15)^.

A number of studies have indicated a positive effect of Telenursing using telephone calls in various mental health conditions such as smoking cessation, anxiety reduction, improvements in depression, reduction of alcohol use and medication adherence among individuals diagnosed with schizophrenia^(16-17)^. However, studies reporting preventive approaches in the mental health area are scarce in the literature. This study was based on studies using Telenursing technologies^(16-17)^, on the intervention program for anxiety^(18)^, and on the application of the Brief Intervention (BI)^(19-20)^.

Telenursing was used to screen and perform an intervention to prevent mental health problems caused by situations of vulnerability such as those resulting from the COVID-19 pandemic, including anxiety symptoms and increased alcohol consumption. The anxiety management program applied in person, in a specialized mental health service, obtained good results in reducing anxiety^(18)^, showing itself as a feasible possibility based on a Nursing theory of adaptation for use through technology, in order to meet the reality of the moment and reach a highly vulnerable population group. With regard to application of the BI, it has been used by Brazilian nurses with similar populations in face-to-face contexts, observing good results^(19-20)^.

The leading role of nurses and Nursing and their importance in coping with disorders related to the use of psychoactive substances has been highlighted by their broad performance in health education, early identification, treatment and rehabilitation of individuals suffering from such disorders^(21)^. These professionals have been pointed out as a key element to face this problem in health systems around the world^(19,22)^, leading the World Health Organization (WHO) to recognize its role in mental health prevention as one of the main achievements of Nursing in recent decades in two consecutive reports (2008-2016 and 2016-2020) of the global strategic guidelines for the strengthening of Nursing and Obstetrics in the world^(23)^. Currently, a paradigm shift is taking place in terms of coping with the use of psychoactive substances; with a transition from a model focused on treating the disorders to focusing on the prevention and identification of harmful substance use. In this context, nurses assume a prominent position mainly in the prevention^(19)^ of disorders related to substance use, highlighting the actions of early identification and brief interventions^(24)^.

Regarding nurses’ performance in the face of anxiety symptoms, a number of systematic reviews^(25-27)^ have shown nurses’ potential to help their patients manage their anxiety symptoms more effectively in different care settings and using multiple approaches^(28)^. With the emergence of COVID-19, the interventions that had already been used by nurses, such as teaching relaxation techniques and breathing exercises among others, had to be reinvented and innovative measures had to be implemented together with the traditional care model^(28-29)^. A recent review pointed out that using Telehealth to teach relaxation techniques, complementary integrative practices (Mindfulness; Meditation and Yoga), education for healthier lifestyle habits and self-guided online psychoeducation were the most used interventions by nurses to manage anxiety symptoms during COVID-19 in health care services around the world. Also according to this review^(29)^ the interventions applied by these professionals, although multiple, showed good results in reducing anxiety symptoms in several populations.

Given the above, this study aimed at investigating the effect of a remote intervention carried out through telephone consultations on anxiety symptoms and alcohol use in users of Primary Health Care (PHC) services during the COVID-19 pandemic.

## Method

### Study design and sample

This is a quasi-experimental study of the before-and-after non-randomized type, conducted with users of four PHC services in the central region of the city of São Paulo. A convenience sample of 1,444 individuals was invited to participate in the study between December 2020 and June 2021. Of these, 1,270 users agreed to take part in the study. Most of the participants were women (n=791; 62.3%) and declared themselves as brown-skinned (n=483; 38.3%) and single (n=488; 38.6%). The mean age of the group was 48.2 ± 16.4 years old.

### Selection criteria

All users over the age of 18, registered in the unit and who had sought health care were invited to participate in the study through telephone calls, according to availability and fulfillment of the eligibility criteria: being over the age of 18; having a record in the health service; having received any health care in the services involved in the last 30 days prior to the telephone contact; being able to communicate in Portuguese, understanding the study description; scoring for moderate, high or severe risk use (score ≥ 3) in the Alcohol Use Disorders Identification Test (AUDIT-C); and/or obtaining scores ≥ 11, indicative of moderate to severe anxiety symptoms in the State-Trait Anxiety Inventory-6 (STAI-6); and being available to undergo follow-up if they were eligible to undergo the interventions.

The exclusion criteria considered the participants’ self-report of being in treatment for problems related to alcohol use and/or mental disorders; presenting noticeable signs of disorientation and mental confusion during the telephone call, which were evaluated by the study team before the beginning of the interview, using questions related to the following information: day and time of the interview, full name, phone number and date of birth; or intoxication by psychoactive substances, adding the evaluation of previous responses to observation of the following phenomena: slurred speech, slowness and/or lack of connection in the answers. In addition to that, undergoing or having undergone psychotherapy in the last 30 days, counseling for anxiety and/or depression, or having participated in mental health interventions, were also considered exclusion criteria.

### Ethical aspects

The research was approved by the Committee of Ethics in Research with Human Beings of the research host institution with Certificate for Presentation of Ethical Appreciation (*Certificado de Apresentação de Apreciação* Ética, CAAE) number 37238720.4.3001.0086. In order to recruit the participants, the schedules of the services involved (four) were obtained through which telephone contacts were made with the possible participants. During the telephone approach, for those who agreed to participate in the study, the Free and Informed Consent Form (FICF) was read aloud, requesting authorization to record the call and emphasizing that all information would be kept confidential. The data were collected by telephone calls and managed via the Research Electronic Data Capture (REDCap) platform.

### Instruments

In order to screen the anxiety symptoms, the State-Trait Anxiety Inventory-6 (STAI-6) scale was employed, validated for use in Brazil^(30)^ in populations from various health contexts and showing good reliability indices (α=0.90). STAI-6 aims at identifying the presence of anxiety symptoms through six statements with multiple answer options from “Certainly not” (1) to “Very much” (4), indicating scores between 6 and 24, with the following cutoff points to classify anxiety: from 6 to 10 = Mild; from 11 to 15 = Moderate; and from 16 to 24 = Severe. To screen the consumption pattern of alcoholic beverages, the brief version of AUDIT (AUDIT-C) was applied in the participating population. This is a simplified instrument validated for use in the Brazilian population^(31)^, with adequate reliability indices (α=0.83) and already applied in different health contexts in Brazil. It has three questions containing five answer options, which allow classifying the use pattern according to gender. For women, scores from 0 to 2 indicates low risk use, while for men this rating corresponds to scores from 0 to 3. Moderate risk use for women is defined by scores between 3 and 5 points and, for men, from 4 to 5; high risk use from 6 to 7 for both women and men and, likewise, severe risk use between 8 and 12 points^(31)^. Finally, a sociodemographic data form was applied containing questions about gender, race/skin color, income, schooling, reason for consultation, having been diagnosed with COVID-19 or having lost someone close with that diagnosis.

### Interventions

Individuals who scored ≥ 3 on AUDIT-C and/or ≥ 11 on STAI-6, suggestive of moderate, high or severe risk alcohol use and moderate/severe anxiety symptoms, respectively, received the Brief intervention and/or the Brief Interpersonal Relationships intervention for anxiety, delivered by a team of nurses and Nursing students previously trained for its application. The brief intervention is a strategy that aims at motivating individuals at risk of substance abuse to change their behavior. It consists of six elements identified by the FRAMES acronym, originated by combining the first letter of the following words: Feedback; Responsibility; Advice; Options Menu; Empathy and Self-efficacy^(32)^. The intervention was initiated with feedback on the meaning of the score obtained by each individual in AUDIT-C and, subsequently, after asking if he/she would like to reduce the risks to his/her health caused by drinking, pointing out, if so, that the participant was the only one who could decide on changes in their actions (Responsibility), the person was advised on harm reduction strategies when indulging in alcohol consumption, such as eating well before and, if possible, during alcohol intake (Advice); followed by a discussion on ab Options menu to avoid situations that predisposed the interviewee to higher alcohol consumption (Options Menu). Every intervention was permeated by the intervener’s empathic stance (Empathy), ending with reinforcement of the participant’s ability to undertake changes in favor of improving their health (Self-efficacy). In the Interpersonal Relationships in Anxiety Situations (IRA), an intervention based on the Interpersonal Relationships in Nursing consisting of 5 elements, it was jointly sought that the individual became aware of the anxiety symptoms; naming anxiety; identification of healthy and harmful anxiety relief behaviors that the individual could be using; identification of the triggers that generated anxiety; and guidance on a menu of healthy anxiety relief behaviors^(18)^.

### Data collection

**Figure 1 -:**
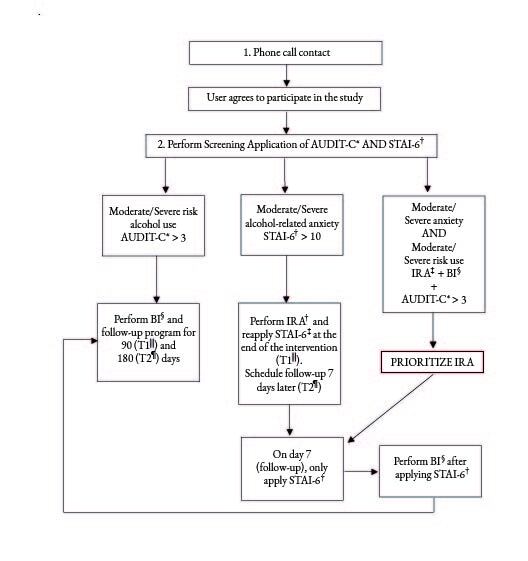
Flowchart corresponding to the data collection procedures

^*^AUDIT-C = Alcohol Use Disorders Identification Test-C; ^†^STAI-6 = State-Trait Anxiety Inventory-6; ^‡^IRA = Interpersonal Relationships in Anxiety; ^§^BI = Brief Intervention; ^||^T_1_= Time 1; ^¶^T_2_ = Time 2

The data were collected among users who sought health care regardless of the reason, in the study scenarios. Each of the interventions was delivered immediately after identifying moderate/severe anxiety symptoms or moderate/severe risk alcohol use with a mean duration of 25 minutes in each intervention. In the cases where the participants presented concurrence of moderate/severe anxiety symptoms and moderate/severe risk alcohol use, the intervention for the anxiety symptoms was prioritized and the Brief Intervention (BI) was carried out later on with one-week intervals between them (Figure1).

Follow-up for those who received the IRA (Interpersonal Relationships in Anxiety), it was initiated immediately at the end of the intervention (T_1_) and the second follow-up (T_2_) was carried out 7 days after its application. For the participants who received the BI, the follow-up was carried out through a new telephone contact with AUDIT-C reapplication after 90 (T_1_) and 180 (T_2_) days of the initial screening and/or application of the brief intervention. Figure1 illustrates the study data collection and follow-up process. In order to minimize the response biases, in all cases, the follow-up was performed by an individual different from that one in charge of screening and the intervention.

### Data treatment and analysis

For data analysis, the categorical variables were described in percentages and the numerical variables by means of central tendency measures. To evaluate the effect of the interventions on the alcohol use pattern and anxiety symptoms, the mixed-effects model was used to compare measures with an additional sequence of two-by-two sequential comparison. The significance level (p-value=0.05) was adopted with a 95% confidence interval.

## Results

**Figure 2 -:**
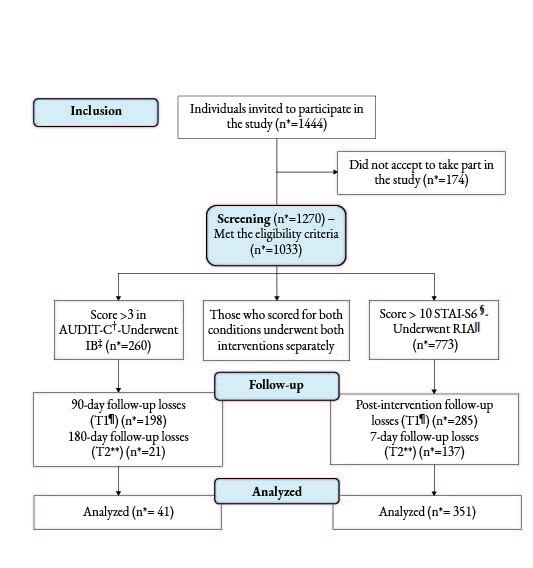
Flowchart corresponding to inclusion of the participants

^*^n = Sample size; ^†^AUDIT-C = Alcohol Use Disorders Identification Test-C; ^‡^BI = Brief Intervention; ^§^STAI-6 = State-Trait Anxiety Inventory-6; ^||^IRA = Interpersonal Relationships in Anxiety; ^¶^T_1_ = Time 1; ^**^T_2_ = Time 2

In order to investigate the effect of a remote intervention on anxiety symptoms and alcohol consumptions in users of PHC services, we had 1,033 PHC users who met the eligibility criteria to participate in the study and, among them, 507 (39.4%) participants obtained a scores indicative of moderate anxiety symptoms and 266 (20.7%) of severe anxiety symptoms. Regarding alcohol consumption, 95 (36.5%) participants scored on AUDIT-C with values suggestive of moderate risk use, 86 (33%) classified as high risk use and 79 (30.4%) as severe risk use. Therefore, at the beginning of the study we obtained 1,033 participants; among which 260 (25%) scored > 3 on AUDIT-C and underwent the BI and 773 (74.83%) scored >10 on STAI-6 and underwent IRA (Figure2).

Of the 773 participants who received the intervention for anxiety (IRA), only 351 (45.4%) completed the study, representing a loss of approximately 50% of the sample (Figure2). However, the results suggest a positive effect of IRA in reducing anxiety symptoms among the participants; there were statistically significant differences between the application moments of the STAI-6 scale, with a mean reduction of 0.5 points at T_1_; however, there was no change in the scores at T_2_ (Table1). These results are consistent with those presented in Table2, which indicate that the difference between the study moments was significantly higher between T_0_ and T_1_, which is not observed in the comparison to T_2_.

**Table 1 -:** Result of the Mixed-effects model for comparison of all three STAI-6^*^ measures: baseline (T_0_^†^), T_1_^‡^ and T_2_[^§^]{dir=“rtl”} referring to IRA^||^ for anxiety. São Paulo, SP, Brazil, 2021

Time	N^¶^	Mean	SD**	*p*-value^††^
T_0_^†^	773	14.8	3.04	<0.001
T_1_^‡^	488	13.1	3.12	
T_2_^§^	351	13.2	3.69	

^*^STAI-6 = State-Trait Anxiety Inventory-6; ^†^T_0_ = Time 0-Baseline; ^‡^T_1_ = Post-intervention follow-up; ^§^T_2_ = 7-day follow-up; ^||^IRA = Interpersonal Relationships in Anxiety; ^¶^N = Sample size; ^**^SD = Standard Deviation; **††** *p*-value = Mixed-effects model

**Table 2 -:** Sequential two-by-two comparison of all three measurements: baseline, T_1_^*^ and T_2_^†^, referring to IRA^‡^ for anxiety. São Paulo, SP, Brazil, 2021

Time/Intervention	Parameter	*p*-value
STAI-6^§^ baseline^¶^ *versus* T_1_^*^	-1.496	0.001
STAI-6^§^ T_1_^*^ *versus* STAI-6^§^ T_2_^†^	0.083	0.842

^*^T_1_ = Post-intervention follow-up; ^†^T_2_ = 7-day follow-up; ^¶^T_0_ = Time 0; ^‡^IRA = Interpersonal Relationships in Anxiety; ^§^STAI-6 = State-Trait Anxiety Inventory-6

With regard to alcohol use, 260 (25%) participants underwent the brief intervention, of which 41 (15.8%) completed the study, characterizing a loss of approximately 80% of the sample (Figure2). Table3 shows the measurements performed T_0_, T_1_ and T_2_ in order to evaluate the effect of the brief intervention in reducing the alcohol use pattern. There is a reduction between the mean of T_0_ and T_1_, equivalent to 1.57 points in the AUDIT-C score. In the two-by-two comparison, there is evidence of a new reduction between T_1_ and T_2_, suggesting that the effect of the intervention in reducing alcohol use takes longer to happen.

**Table 3 -:** Mixed-effects model to evaluate the effect of the brief intervention on the alcohol use pattern, comparing all three AUDIT-C^*^ measures: T_0_^†^, T_1_^‡^ and T_2_^§^. São Paulo, SP, Brazil, 2021

Time	N^||^	Mean	SD^¶^	p-value
T_0_^†^	260	5.69	2.38	<0.001
T_1_^‡^	62	5.00	3.01	
T_2_^§^	41	4.12	2.71	

^*^AUDIT-C = Alcohol Use Disorders Identification Test; ^†^T_0_ = Baseline; ^‡^T_1_ = After 90 days; ^§^T_2_ = After 180 days; ^||^N = Sample size; ^¶^SD = Standard Deviation

## Discussion

The COVID-19 pandemic has imposed multiple consequences, not only to the physical health but also to the mental health of the population, which has been documented by several authors^(33-36)^ who claim an increase in depression^(23)^, anxiety, obsessive-compulsive and post-traumatic^(37)^ symptoms related to a variety of negative emotions, such as fear of dying and panic of being or remaining locked in the house, among others that have not been adequately processed by the population^(37)^. The results obtained in this study are consistent with the literature, indicating a representative number of individuals with anxiety symptoms between moderate and severe, which deserves attention in different health scenarios, including Primary Health Care units.

In addition to that, fewer people with risk alcohol use were found when compared to those who manifested anxiety symptoms. This can be related to difficulties already mentioned in the literature, some of them linked to gender. A number of studies indicate that women are afraid of feeling judged^(38)^ when talking about the use of psychoactive substances and, considering that the sample was mostly female, it is likely that the female users did not feel confident to provide data more consistent with the reality of alcohol consumption. On the other hand, the research was developed via telephone calls, which makes it a challenge to establish the link between researcher and users, as well as an environment where the individual feels truly welcomed^(39)^.

Faced with this situation, creativity and the search for remote care strategies to overcome such challenges imposed by the distancing measures began to occupy the agenda of health systems in different care contexts. Technology use allowed providing necessary and fair care services to the patients who were at their homes and did not have access to some specific services^(37)^; however, health professionals had to develop innovative skills/competencies to ensure care quality, safety and efficiency through technology^(40)^. The current study meets this proposal, aiming to evaluate two psychosocial interventions applied remotely by Nursing professionals in the Primary Health Care context.

The interventions proposed in this study proved to be initially effective in reducing anxiety symptoms and risk alcohol use, also pointing out that they can be employed by nurses and that they have the potential to reach a large percentage of the population, overcoming the digital poverty barrier and being useful in situations where accessibility of the users or professionals is compromised^(3)^. There is evidence^(11)^ for the use of Telenursing interventions and their potential for the management of anxiety symptoms related to isolation and the sensation of confinement during the pandemic. The users have also been receptive to this technology, which can be considered a possibility of approaching and welcoming in the different health services, through basic low-cost tools such as telephone and Internet connections^(12)^, rendering the intervention feasible for most patients.

Brazil has been one of the most affected countries by the pandemic, a situation that requires responses to the mental health demands of the population; it is likely that important changes occurred in the post-pandemic period, especially in the way in which we worked before COVID-19, so there is a need to think about integrative approaches to the Nursing practice, with Telenursing in mental health among them. Telehealth has been part of the Brazilian health system since 2007, gaining major prominence during the COVID-19 pandemic. There are studies^(41-42)^ documenting its use in Brazilian Nursing in the last two years, due to the health contingency^(43)^. The published studies that correspond to this period are mostly related to respiratory care and physiological complications of COVID-19^(44-45)^, care for older adults^(46)^ or maternal and child health^(42)^, which makes our study innovative when exploring a useful tool for mental health Nursing care in the post-pandemic period.

The mental health Telenursing presented in this study highlights the possibility for nurses to appropriate technologies inherent to their practice, such as the interpersonal relationship in Nursing^(47)^ and the helping relationship^(48)^, to enable people to identify the triggers and aggravating factors for their anxiety symptoms and other situations that cause psychological distress.

With the instrumentation to recognize mechanisms for coping with their symptoms, subjects are able to manage such symptoms, recognizing their aggravating factors and providing changes for their reduction. Furthermore, in addition to coping with conditions related to mental health, telephone calls in catastrophes and disasters situations have the potential to mitigate the sensation of isolation and helplessness that may be experienced by the population, especially those who are more vulnerable and who enjoy less access to digital technologies. Finally, it is necessary to consider that, although remote mental health care can be a valuable possibility with countless potentialities for improvement, expansion and use in the specialty, it does not replace the usual care that strives for integral communication and person/person contact^(47)^.

This study has some limitations, such as the methodology selected, which did not allow comparisons with the Control Group for being a quasi-experimental approach. However, studies of the clinical trial type, both randomized and pragmatic, should be prioritized in order to enable more data on the effectiveness of the intervention proposal herein presented.

Regarding the contributions to the Nursing area, this study stands out for presenting the proposal of a program of phone calls made by nurses as a resource for remote mental health care, for people who have common mental disorders such as anxiety symptoms and harmful alcohol consumption in cases where face-to-face monitoring is not possible or involves some type of risk for the user and/or the health professional. In addition to that, telephone consultations can be more economical, simple and convenient even for the patients, as most aged people who have limited mobility are not familiar with remote audiovisual communication, turning it into a possibility that may occupy privileged place in a digitized society and ensure safety, quality and equality in health standards.

## Conclusion

The results suggest a positive effect of our intervention in reducing anxiety and the alcohol use pattern. In addition to the benefits of the Nursing intervention to prevent worsening of mental health conditions, the intervention suggested is an alternative to reach even those with fewer digital resources and without Internet access, as a large part of the country’s population does not have access to the network. In these cases, telephone calls can offer a low-cost, convenient and methodologically simple service for delivering information on health, education and psychosocial support to different population groups and strata.
